# Leading higher education into the fourth industrial revolution: an empirical investigation

**DOI:** 10.3389/fpsyg.2023.1242835

**Published:** 2023-08-30

**Authors:** Shwetha Singaram, Claude-Hélène Mayer, Rudolf M. Oosthuizen

**Affiliations:** ^1^Department of Industrial Psychology and People Management, College of Business and Economics, University of Johannesburg, Johannesburg, South Africa; ^2^Department of Industrial and Organisational Psychology, University of South Africa, Pretoria, South Africa

**Keywords:** fourth industrial revolution (4IR), technology, higher education, leaders, skills

## Abstract

The Fourth Industrial Revolution (4IR) rapidly advanced at the beginning of the 21st century. Leaders within organisations need to adjust their visions, plans, organisational structures, and management with regard to the demands, challenges and opportunities of this development. This is in particular the case in higher educational institutions (HEIs), which have to adjust to the rapid changes and new demands of skills of university graduates. Leaders in HEIs must therefore be aware of the related challenges and opportunities and might have to adjust the learning and teaching environment, the skills development of students, graduates, and faculty, as well as the technological requirements to create advanced skill sets. This article is based on a qualitative research study which was conducted at a university in South Africa. In-depth, semi-structured interviews were used to explore the views of higher-education leaders at the selected university. Data were analysed through thematic analysis. It was found that leaders in HEIs need to be aware of their leadership and preferences in times of transition toward a more technologised learning environment, as well as the needs, demands, challenges and opportunities of the new workplaces, and new skill sets needed in the 4IR. The researchers made some recommendations.

“Education is the key that unlocks the golden door to freedom.” *George Washington Carver.*

## 1. Introduction

The Fourth Industrial Revolution (4IR) has become a buzz word over the past number of years (Mhlanga and Moloi, 2020), although its meaning differs in different cultural contexts (Tondi, 2019). The 4IR is defined as a time period that changes the world radically through new technological advancements while uniting digital, physical and biological domains (David et al., 2022) and implementing shifts in workplaces, wealth, power and knowledge (Xu et al., 2018).

Generally, the 4IR has been conceptualized as a new way of living, communicating, working, and using technology (Schwab, 2017). Even though the 4IR poses significant social and economic challenges and threatens employment opportunities, Schwab (2017) urges everyone to embrace the chance to shape and guide it toward a future that represents society’s universal objectives and values. Technology affects all societies and alters economies and socio-cultural environments in an all-inclusive and universal way (Qazi et al., 2020; Anshari et al., 2022). Organizations often do not seem to adhere to a specific theoretical framework of the 4IR (Newman, 2019; Mhlanga, 2022). The literature on understanding and shaping the application of the 4IR is overwhelmingly focused on Western or developed contexts, since developed countries have greater technological access and experience than developing countries (Kerres, 2020). Therefore, this research contributes a sociocultural perspective on understanding and shaping 4IR in a South African context, that is, the context of a developing country.

The 4IR has been studied internationally and attitudes have been found to play a crucial role in understanding and implementing it. Additionally, according to Ślusarczyk (2018), most business leaders are optimistic about the 4IR’s potential to develop and improve competitiveness among businesses. Compared to international universities, for example in Singapore (Gleason, 2018), there is only minor research in the South African context on how HEIs and leaders in HEIs are responding to the 4IR, even though it is essential to consider the culture-specific context of the 4IR. To contribute to a holistic perspective of what the 4IR may mean to leaders in HEIs in South Africa (Shaturaev, 2022), this research aims to examine leaders’ views on the 4IR in a selected higher-education institution in South Africa.

The study explores leadership in the HEI in South Africa and is thereby focused on a specific cultural context with regard to the country, the organization and the cultural environment of the 4IR. Culture is defined broadly in this article as the way people, including leaders as well as employees, do things around the organization (Schein, 2004).

The aim of this article is to explore the leaders’ perspectives on the challenges and opportunities the 4IR poses for higher education in South Africa. The research question responding to this aim is: on what do leaders in a specific HEI focus to respond to the demands of the 4IR in South Africa in terms of the emerging challenges and opportunities?

## 2. Higher education in the fourth industrial revolution

The education sector has cautiously applied 4IR technology to transform teaching and learning practices (Mhlanga and Moloi, 2020). As the 4IR unfolds, the education sector will face both opportunities and challenges in terms of curricula, teaching, and learning (Kayembe and Nel, 2019; Mbandlwa, 2021; Chaka, 2023). The 4IR has begun to transform society’s functioning since the beginning of the 21st century, but its full use in teaching and learning has been limited to supporting traditional teaching and learning methods in the form of project materials and shared learning on virtual platforms (Oke and Fernandes, 2020; Modise and Van den Berg, 2021). Many factors contribute to this limited use, including ignorance or limited access to technological advances, comfort with traditional approaches, and recent concerns and questions about 4IR technology’s ethical, pedagogical and epistemological implications (Mhlanga and Moloi, 2020). Taking advantage of the different technologies required by 4IR in higher education requires the appropriate skills (Kayembe and Nel, 2019). Students have to be digitally literate to adapt and survive in a digital world (Rusdah and Sutarsih, 2021).

The use of 4IR and associated technologies in manufacturing has been extensively studied, but there are fewer studies exploring the perspectives of 4IR among staff and students in African education (Oke and Fernandes, 2020; Mbandlwa, 2021). It is this gap that limits the use of 4IR technologies in education and their impact on stakeholders (Maj-Waśniowska et al., 2023). A study conducted by Ng’ambi et al. (2016) suggests that South Africa has not reached that level of innovation or collaboration within the government yet. Ng’ambi et al. (2016) reviewed the literature of technology-enhanced teaching and learning in South African higher education over the last 20 years (1996–2016).

Further research focused on pedagogy enabled by technology, the digital divide, and epistemological access. On an international scale, social networking and advanced computing are being explored (Ng’ambi et al., 2016). To meet the new demands of the 4IR era, teaching and learning at HEIs need to be adapted to better prepare the emerging workforce (Penprase, 2018; Modise and Van den Berg, 2021).

Between 2011 and 2016, South African universities began using social media and mobile learning platforms, as well as introducing digital devices. There was, however, wide variation in utilization and access across South African HEIs, with some having greater access and resources and making greater progress than others (Ng’ambi et al., 2016). The South African HEI sector still lacked a comprehensive national strategy document or policy on the role of information and communication technologies (ICTs). Indeed, Ng’ambi et al. (2016) found that South African HEIs have limitations in the development of technology-enabled higher education. As a result of poverty and political discrimination, there is unequal access to resources and education.

## 3. Higher education context during the 4IR and COVID-19

First reported in December 2019, the COVID-19 virus was declared a pandemic by the World Health Organization in March 2020 (Qazi et al., 2020). Globally, governments implemented lockdowns to reduce human contact to curb the spread of the virus (Mishra et al., 2020). Students and scholars were able to continue their academic year by using online learning systems (Ali, 2020; Kerres, 2020). According to UNESCO (Oyediran et al., 2020), by utilizing online lectures, e-textbooks, virtual classrooms, and communication platforms, the COVID-19 pandemic led to the rapid digitisation of the education system in South Africa (Qazi et al., 2020). HEIs were developing strategies to ensure a smooth uptake of the new technologies, including training and support for their members, but little consideration was given to how institutions and diverse stakeholders would respond and cope (Kerres, 2020).

Globally, the COVID-19 pandemic revealed several vulnerabilities in educational systems, such as an underdeveloped online teaching infrastructure, unskilled educators, a complex home environment, and information gaps (Ali, 2020). As a result, education systems needed to be resilient and adaptable (Aslani and Jacob, 2023). Online learning systems were introduced, and research has shown that, in addition to technology-enabled resources, staff readiness, accessibility and motivation are all important factors in optimizing the uptake of online and digitized education (Ali, 2020). It is important to note that the adoption of online education was beneficial and considered to be a “lifeline for education during the pandemic” (Oyediran et al., 2020). To guarantee that education and learning continued during the COVID-19 lockdown, China developed a policy called ‘Suspending Classes Without Stopping Learning’ (Oyediran et al., 2020).

Because of poor online educational infrastructure and unequal access to the internet and technological devices, digitized education presents a unique challenge to many African countries’ teaching and learning development systems (Suganya, 2017; Oyediran et al., 2020). In the Nigerian educational context, the high cost of technology accessories and a lack of digital resources are cited as obstacles to online learning systems (Oyediran et al., 2020). According to Penprase (2018), a 4IR educational strategy should take into consideration how it will affect people from various socio-economic backgrounds, maintain human rights, and develop an understanding of society’s transformation on an intercultural level. According to Xing et al. (2018), South African HEIs will benefit from and drive the 4IR in the country. A number of South African HEIs are expected to implement and integrate 4IR technology within their institutions, using technology to bridge the gap between students (Xing et al., 2018) and build an innovation-based, supportive education culture (Lee and Yuan, 2018).

## 4. Critical skills, problem solving, collaboration and adaptability in higher education 4.0

The concept of Higher Education 4.0, developed in consultation with leading education experts from both the public and private sectors, could increase global productivity tremendously (Advani, 2023). Three key areas have been identified for investing in HEIs to unlock this transformation within the 4IR: new assessment mechanisms, new learning technologies, and teacher empowerment (Maj-Waśniowska et al., 2023). Educators, businesses, investors, parents, and caregivers, as well as students themselves, all have a role to play in realizing the promise of Higher Education 4.0.

Advani (2023) postulates that a key component of Higher Education 4.0 in South Africa will be to reimagine higher education as a lifelong experience that places the responsibility **for skill-building** on the student, with educators and mentors acting as facilitators and enablers. Investing in new HEIs and upgrading existing HEIs should be a part of the education strategy. Educators, parents and the business community should work together to prepare students for the future (Chaka, 2023).

Three skills have been identified in the literature as being relevant skills that need to be addressed and fostered in the Higher Education 4.0 context to prepare the future workforce for the 4IR challenges: problem-solving, collaboration and adaptation (Advani, 2023). They will be needed to deal with the complexity of the 4IR, as defined above.

**Problem-solving** skills can be developed in students who are curious about the challenges they face and who are ready to embrace them (González-Pérez and Ramírez-Montoya, 2022). By relying and building on creativity, data analysis, perseverance, and critical thinking, students will develop the problem-solving skills needed for the 4IR (Advani, 2023).

**Collaboration** in Higher Education 4.0 involves working well with others. Students who are collaborative are influenced by good data and effective persuasion, and they are willing to change their minds if confronted with contrary evidence (Advani, 2023). A successful collaborator builds relationships with individuals of all personality types, work styles and backgrounds, resolving conflicts within a team quickly and efficiently. Communication is respectful, whether it takes place in person, on camera, via audio, when writing in any form (from a few words to a lengthy report) or when listening actively (Moraes et al., 2022). Pearson Education reviewed findings on how to teach students to collaborate with the Partnership for 21st Century Learning (González-Pérez and Ramírez-Montoya, 2022; Advani, 2023). Three elements of collaboration should be incorporated into everyday classroom activities: interpersonal communication, conflict resolution, and task management. To develop and practice collaboration skills, a certain degree of friction must be incorporated into the learning environment (Reimers and Marmolejo, 2022).

An individual’s **ability to adapt** ranges from a certain level of comfort with uncertainty, sudden changes and unfamiliar circumstances, to the ability to develop innovative solutions in pressured circumstances (Venkatraman et al., 2022). When students are adaptable, they can transition seamlessly between following and leading. To improve themselves, such students enjoy learning new skills, mastering new subjects, and testing themselves (Advani, 2023).

Learning to adapt develops resilience, buoyancy, and self-regulation, and requires cognitive, behavioral and affective (emotional) adjustments. Teaching adaptability involves creating a self-regulatory process for students that involves them self-evaluating their proficiency in a particular area, establishing learning goals, building experience and skills, evaluating proficiency again and identifying the modifications that need to be made to continue improving (Prada et al., 2022). The ability to adapt and modify skills and behaviors over time is developed through evaluation and feedback (Advani, 2023).

## 5. Research methodology

A qualitative, post-modernist research design with a holistic, in-depth understanding of rich, contextual information, which cannot be generalized (Queirós et al., 2017), was used for this study (Ponelis, 2015). An interpretivist paradigm, which assumed that reality is constructed within the selected organization, was applied. The objective was to investigate the perspectives of the participants to discover their worldviews (Ponelis, 2015; Thanh and Thanh, 2015). The study further sought to understand the socially created reality and social practice used in the context (Hyde, 2020).

Phenomenology was adopted as a research strategy, in line with the qualitative design of the study and the philosophical assumptions to be applied. To better understand people’s lived experiences of a phenomenon, phenomenological research is considered a valuable method, as it aims to describe, understand and decipher the meaning of a phenomenon through the perspectives and experiences of those who have experienced it (Marques and McCall, 2005; Neubauer et al., 2019). As part of this research strategy, detailed descriptions were obtained from participants through in-depth interviews, and these were then condensed into common themes and descriptors explaining the phenomenon based on the participants’ experiences (Marques and McCall, 2005). Through phenomenology, we can gain insight into participants’ experiences and how they perceived them (Zolnikov and Furio, 2020).

### 5.1. The context and sample

In this study, non-probability sampling was applied, and participants working as academics and in management in the College of Business and Economics at a selected university in Gauteng were purposively approached. The study used the homogenous sub-type of purposive sampling method, whereby the participants are selected based on their similar characteristics (Etikan et al., 2016). The participants shared the attributes of being employed by the same university in Gauteng and belonging to the same faculty.

The sampling process was conducted with regard to the saturation of the data, and this was the case when seven interviewees were interviewed (Fusch and Ness, 2015; Farrugia, 2019). The sample consisted of two senior lecturers, one Head of Department, one Deputy Head of Department and three participants who made up the Deanery for the College of Business and Economics. In terms of gender, two individuals defined themselves as female, five defined themselves as male. The inclusion criteria were as follows: (a) permanent staff members for a minimum of six months, (b) who are over the age of 18 years.

The university is considered a complex organization because of its dual function of sharing knowledge for the benefit of scholars seeking education, as well as producing knowledge that meets the changing demands of society and industry (Gaus et al., 2019). To obtain a deeper understanding of how the 4IR is understood by academics and leaders and how the principles of the 4IR are reflected in the organization, the researcher used a qualitative research approach and collected data from semi-structured interviews.

### 5.2. Data collection and data analysis

Primary data was collected using semi-structured interviews, which is a qualitative data-collection technique. Using interviews is an effective way to examine participants’ experiences, thoughts, and principles (Gaus et al., 2019) and provide participants with a way to express their subjective perspectives (Dearnley, 2005; Evans, 2018).

The semi-structured interviews were conducted electronically with the seven participants. The interviews were transcribed verbatim, and data were stored safely on a password-protected computer. This type of qualitative data is traditionally collected through in-person interviews (Evans, 2018; Archibald et al., 2019; Gray et al., 2020). However, the researchers conducted the interviews virtually, using the Zoom platform, owing to lockdown restrictions imposed in South Africa during the COVID-19 pandemic.

Braun and Clarke’s (2012) thematic analysis were used to analyse the qualitative research data. Thematic analysis involves identifying patterns of meaning within a data set, organizing them, and providing insights into them (Braun and Clarke, 2012). Since thematic analysis involves core skills that can be applied to other qualitative research methods, Braun and Clarke (2012) recommend it as a foundational method for qualitative data analysis. The researcher is the instrument for thematic analysis, analysing the data and deciding what to code, theme, and contextualize (Nowell et al., 2017). A thematic analysis follows six steps or phases, which were carefully followed and applied in this study. The process begins with the researchers familiarizing themselves with the data through reading and re-reading through the seven transcripts, then assigning preliminary codes to define the data, grouping similar codes into themes, enhancing the themes according to coded data pieces to ensure the meaning of the data is accurately captured, defining and naming the themes, and lastly, reporting on the themes (Braun and Clarke, 2012). The researchers’ application of this process is described further below.

Using Microsoft Excel and Word, the researchers made notes and comments about the data. The data analysis was conducted without the use of any additional software. Transcription was needed to make the interview data more usable for analysis (Castleberry and Nolen, 2018). Following a systematic review of the interview transcripts, the researchers developed initial codes to describe the data (Braun and Clarke, 2012) by highlighting specific sections of the data that provided deep insight and aligned with the study’s objectives and research questions. Researchers actively interact with the data and use qualitative coding to integrate large amounts of data into a meaningful format (Nowell et al., 2017). A Microsoft Excel codebook was created to record the identified codes and their justifications. To answer the research questions, the codes were grouped according to similarity (Braun and Clarke, 2012). Experiences (codes) that might seem meaningless by themselves have meaning when woven into the themes (Castleberry and Nolen, 2018). By using inductive analysis, the researchers identified the themes explicitly from the data, rather than taking them from pre-existing studies (Braun and Clarke, 2012). The themes were then re-examined against the data codes to ensure that they formed a comparable pattern, to identify any inaccuracies in the coding, and to determine if the data collected provided adequate evidence to support the identified theme (Braun and Clarke, 2012; Castleberry and Nolen, 2018). The meanings generated by the study are enhanced when the research output is linked to the wider literature. Direct quotes from the participants are included (Castleberry and Nolen, 2018).

### 5.3. Quality criteria and ethical considerations

To determine the reliability and validity of a qualitative research study, Connelly (2016) developed four criteria for assessing its data, interpretation and methods and identified the four criteria of reliability, confirmability, credibility, and transferability. The trustworthiness of qualitative research affects readers’ and other researchers’ confidence in the data and outcomes of the research (Cypress, 2017). A study’s credibility is determined by the degree of truth or validity of its findings (Yadav, 2022). The researchers aimed to assure that the interpretations of the data were accurate and as much as possible free from individual bias. The participants’ responses were securely stored to prevent data corruption or tampering. By documenting how the study was conducted and reporting the research findings, the researchers ensured the reliability of the study (Noble and Smith, 2015).

Data and interpretations made in a study are presumed to be confirmable if there is a logical process that can be repeated (Cypress, 2017). Documentation of each step of the data-collection and analysis process, including interview notes, transcripts, recordings, and a codebook, met this criterion. The study’s findings are related to the relevant literature, and detailed descriptions are given. Transferability refers to how well the study’s findings can be used in other contexts or by other researchers (Connelly, 2016; Yadav, 2022). To ensure transferability, the researcher provided detailed descriptions of the research process, sample and context, as well as the study’s relation to existing literature. This impacts the quality of the research study and the reliability of the responses obtained (Swain, 2016; Feldman and Shaw, 2019).

Based on the ethical principles of autonomy, beneficence and nonmaleficence (Stake, 2005), informed consent and voluntary participation were required, and confidentiality was ensured (Mumford, 2018). Voluntary participation contributes to the integrity of the responses of participants by removing external pressures or stimuli (Kılınç and Fırat, 2017). It has always been the responsibility of psychologists to protect the confidentiality of individuals as part of their core values and responsibilities, to protect the people with whom they work (Fisher, 2008). The researchers only had access to a password-protected computer during this study, to ensure the confidentiality of the participants’ responses. The results were reported without identifying information about the participants.

## 6. Findings and discussion

Focusing on the research question “On what do leaders in a specific HEI focus to respond to the demands of the 4IR in South Africa in terms of the emerging challenges and opportunities?” three emerging leadership themes were identified, namely the 4IR technology, the COVID-19 pandemic, and the organizational culture, with the influence of leadership as a common thread. Leaders are highly aware of their role in driving the organization through the 4IR and the challenges of the COVID-19 pandemic, both of which resulted in the urgency to develop an educational system that is increasingly technologised.

### 6.1. Leading through technological changes in the 4IR

This study explored how participants understand the 4IR, and the influence of leadership on its implementation at the university. The study concurs with the literature in that leaders in the HEI see technological change as key to the future of the organization and a chance to develop (as in Schwab, 2017).

The leaders of the organization saw the 4IR as the influx of technology, and this was confirmed by the literature (Anshari et al., 2022). The participants expressed a range of emotions toward it. Some felt excited, while others were fearful or ambivalent. P5 shared, for example, that “the most critical thing about the 4IR is that people have become more innovative in how they address the normal day-to-day problems.”

Critical skills and problem solving are also key educational aspects in HEIs, according to Advani (2023). Although Western societies seem to be more advanced in terms of 4IR technological changes (Kerres, 2020), leaders in South Africa’s HEIs are very aware of the innovative implications of the new technologies and they are on top of the 4IR discourses in the Western and African contexts which aim to unlock the potential of technology, employees, and learners in Higher Education (HE) 4.0 (Maj-Waśniowska et al., 2023).

The participants described how the university is implementing the 4IR, with investments in infrastructure and by incorporating 4IR content into curricula, as described in Chaka (2023). P5 highlighted that “this university is using chatbots to respond to students at all hours,” and that “the 4IR culture and ways of thinking have had an impact on the faculty’s teaching, learning and planning strategies.” Also, Advani (2023) mentions that HE 4.0 enables all stakeholders to build skills through the curriculum. P7 added that the “use of technology for enablement and empowerment has become part of the university’s language and culture.” The participants also referred to the opportunities that technology affords the university to expand its offerings and accessibility – as P2 stated, “it will level the playing field between universities and online course or qualification offerings” (which is part of building a 4IR HEI setting) (Advani, 2023). However, there are some challenges to implementing technology at the university, as pointed out by P1: “Some clarity is still needed on how to apply 4IR in the various spaces and faculties in the university.” P3 added that the “4IR offerings are still a bit disjointed. We need to incorporate 4IR more seamlessly into the DNA of academics.” According to the literature, this integration needs to happen through collaboration, building skills and incorporation in the learning environment (Reimers and Marmolejo, 2022), which still seem to be missing to a certain extent in the HEI.

The primary driver of the university’s 4IR agenda is the current Vice-Chancellor (VC), who is seen as the champion of the 4IR at the university. P6 stated that “people have accepted this drive and the university is making a collective effort to achieve its goals linked to the 4IR.” P5 added that “the VC’s mission and vision found its way through the entire university culture, through the various structures.” The participants indicated that the university leadership’s drive to assimilate 4IR technology and thinking at the university is supported by innovative spaces and the expertise provided by both local and international staff members. Although this has led to significant uptake by staff and students, the participants pointed out the need for the university to consider the impact of the 4IR on inequality in South Africa and to take social responsibility to contribute to closing that gap among its students. P2 shared their concern that “the university is not being critical enough and is embracing the 4IR changes too easily,” and P6 added that “the technology needs to be appropriate to the environment in order for it to succeed.” Overall, the study revealed the significance of leadership’s influence in implementing the 4IR at the university, as well as the need to consider the societal context in the process.

### 6.2. Leading through the COVID-19 pandemic

Qazi et al. (2020) have described the differences in how HEIs dealt with the pandemic. The university leadership’s influence was evident in the university’s swift transition to online learning and the implementation of technology during a time of disruption, in the form of the COVID-19 pandemic. P1 indicated that “COVID-19 accelerated the use of online learning,” and P5 added that “though COVID-19 was a bad thing, it served as a catalyst for us to rethink how we do things, and we are going to do things differently.” The data shows that the university successfully used its existing online platform and provided additional resources, such as data and training to both staff and students, and that “online teaching support has been phenomenal” (P3). This adds to previous research which shows that teaching adaptability improves HE 4.0 (Prada et al., 2022). The pandemic had a strong impact on organizations (Newman, 2019; Mhlanga, 2022) and although 4IR resources were limited for this HEI in South Africa, the leaders of the organization took the pandemic as a key to change in an optimistic way (as in Ślusarczyk, 2018) and increase their competitiveness, while evaluating the challenges and opportunities in the situation (Shaturaev, 2022).

However, the rapid changes significantly impacted staff members, who felt cautious and overwhelmed by the additional workload and the new technology, while having to navigate their personal lives through the pandemic. However, staff and students are required to be even more adaptable (Advani, 2023). The university’s leadership recognized the impact of the pandemic on staff members’ psychological and emotional wellbeing and took steps to address it, such as regularly sending surveys to check on staff members’ wellbeing and coping levels, and communicating with staff about the various changes, in particular the changes in the curricula, teaching and learning that had to be implemented radically (Kayembe and Nel, 2019; Chaka, 2023). Here the needed collaboration between stakeholders was shown (Reimers and Marmolejo, 2022).

P6 shared that leadership tried to anticipate “staff’s resistance to change and tried to think of ways to manage the resistance.” P7 added that leadership found that “conversations and consultations with staff also helped to find meaning from all the changes and processes.” As mentioned in the literature, ignorance of the staff members regarding the implementation of new technologies, missing skills and digital illiteracy, and comfort with traditional approaches (Kayembe and Nel, 2019; Mhlanga and Moloi, 2020; Rusdah and Sutarsih, 2021) were addressed by the leaders of the HEI.

### 6.3. Leadership in constructing the organizational culture

The concept of organizational culture was a prominent topic in the data gathered from participants within the university. The participants described organizational culture as the way in which the organization operates, its practices, relationships, and interactions between members. P5 highlighted that it is a “common thread that permeates the entire organization.” It has been shown that the organizational culture needs to include critical skill development, problem solving and collaboration (Advani, 2023). The participants reflected that the organization’s culture is influenced by beliefs and values and can play a strategic role for organizations by setting them apart and attracting the right talent. P1 stated, “if you have a good organizational culture, you are going to attract the right kind of people.” The “right people” are associated with critical skills and problem-solving (Advani, 2023).

The observable aspects of the organizational culture mentioned by the participants included the university’s research output, physical structure and facilities, communications, and atmosphere. Communication and conflict management are also seen as important aspects of 4IR skills (Prada et al., 2022; Reimers and Marmolejo, 2022). The participants acknowledged that while the university had updated some of its infrastructure such as lecture venues, some areas remained technologically outdated and ill-equipped, which impedes innovative teaching and learning. P1 felt that it is “very frustrating when the technology does not work in the lecture venues and it even disheartens lecturers from adding additional features to their lecture slides, such as videos, because the technology will not support it.”

The participants acknowledged the competitive culture at the university and the pressure felt by staff to maintain a high ranking and significant research outputs. P3 mentioned that the university’s “activities, from research, teaching and community initiatives are aligned to increasing its ranking.” The university’s ambitious and competitive culture, which allows it to reach international standards, was identified as the primary value that influences all members of the organization. With a competitive culture that is based in rankings, the participants identified the continuous communication from leadership as something that helped them focus on the university’s goals and values and how to achieve them. P1 confirmed that the emphasis on driving results pushes staff to “have to maintain a certain momentum.” P4 mentioned that the different “forms of communication definitely give me a clear line of sight in terms of what’s required of what I need to do and how my goals or my contribution fits into the bigger picture.”

This finding supports the idea of Prada et al. (2022) that goals needed to be established and experienced, skills needed to be built and behaviors had to be developed and evaluated. Feedback also needs to be given to develop goals and values further (Advani, 2023). The findings also showed that, in addition to regular communication, the participants recognized other contributions made by the university’s leadership, especially the Vice-Chancellor (VC), in driving the organization’s goals and culture. The participants mentioned how each VC focuses on a specific agenda and that this significantly impacts the direction of the university’s development, research, and strategic outputs. The current leadership of the university has directed the university’s efforts toward technological innovation and the 4IR and is thus part of the global trend of HE 4.0. P2 confirmed that the “deputy Vice-Chancellor for research is very much involved in the 4IR sort of domain and agenda and they are pushing for that.”

The findings of this study also suggested that the leadership played a significant role in the university’s response to the pandemic, particularly in its successful transition to online learning. However, the rapid changes also had a significant impact on staff members, highlighting the need for ongoing support and resources from leadership, which has been described in previous studies (Maj-Waśniowska et al., 2023). The participants shared a range of emotions about the 4IR and its implementation at the university, which was accelerated by the COVID-19 pandemic. The COVID-19 pandemic had disrupted the university’s operations and the university leadership’s influence is evident in how the university had successfully responded to the crisis, including ensuring the wellbeing of staff and students while transitioning to online learning. Overall, the study emphasizes the significance of leadership’s influence in shaping an organization’s culture, implementing new technology, and effectively responding to disruptions.

As seen in previous studies (Ng’ambi et al., 2016), leaders have not highlighted cooperation with the government in South Africa, which might contribute to an improvement in HEIs and the implementation of 4IR technologies, nor have they mentioned a national strategy that needs to be outlined. The leaders seemed to be relatively self-sufficient regarding implementing their own changes in the HEI, while preparing staff members for the upcoming changes (as in Penprase, 2018; Modise and Van den Berg, 2021). The university’s competitive culture and the current leadership’s focus on technological innovation and the 4IR has significantly impacted the direction of the university’s development and research outputs.

## 7. Conclusions and recommendations

The main question this study aimed at responding to was, “On what do leaders in a specific HEI focus to respond to the demands of the 4IR in South Africa in terms of the occurring challenges and opportunities?” This study therefore contributes to filling the void of research on HEIs and African education and the 4IR (Oke and Fernandes, 2020; Mbandlwa, 2021).

The study showed that that leaders in a selected HEI in South Africa were going with the international trend of implementing 4IR changes within the organization. They emphasized that strong leadership and knowledge about technological changes in the world are of major importance when leading employees, as well as other stakeholders such as students and parents, into the educational future. Leaders also seemed to go with the ideas of leading economic and educational individuals in the field. They are changing curricula, incorporating 4IR content, building in specific skills development and fostering an innovative HEI culture. Further, they are constructing a critical digitisation component within their universities and emphasizing the importance of making a collective effort in developing innovative HEI spaces. They are aware that the pandemic pushed leaders and employees toward a rapid shift and that, during times of such radical change, leaders needed to work to manage resistance and focus on the mental health and well-being of employees. They therefore aim at building a constructive, innovative, and critical organizational culture which is based on cooperation. However, the findings showed that besides the appreciated competitiveness, the cooperation between all stakeholders could still improve, as well as the basic technological support. These will be crucial leadership issues that would need work in the future to reach the top international standard of being an HE 4.0 institution.

This study is based on the perspectives of purposively selected academic staff members within a specific faculty at the university, which places a certain limit on the study’s findings to the experience and perspectives of the selected sample. Thus, further research may be conducted to collect the perspectives of other staff and even students across the university, in the various faculties. The study used a qualitative methodology to interview the seven participants for deeper insights. However, combining the semi-structured interview strategy with a semi-structured or structured online survey may be helpful in increasing the sample size, especially since the data was collected during a strict COVID-19 lockdown. It will also be useful to conduct further research in the current context, to investigate and compare the university’s uptake of technology with their leadership’s influence, post the COVID-19 pandemic.

A recommendation for future research is the study of constructing best practices for HE 4.0 institutions to improve individual, organizational and societal developments, to support 4IR changes. On a practical note, leaders, and HE 4.0 institutions need to focus especially on global, international and internal organizational cooperation and development to drive technological change, critical skills, talent management and an innovate culture for a successful future.

## Data availability statement

The raw data supporting the conclusions of this article will be made available by the authors, without undue reservation.

## Ethics statement

The studies involving humans were approved by the University of Johannesburg, College of Business and Economics Ethics Committee. The studies were conducted in accordance with the local legislation and institutional requirements. The participants provided their written informed consent to participate in this study.

## Author contributions

SS collected the data. C-HM supervised the work. All authors contributed to the article and approved the submitted version.

## Funding

The research project was funded by the National Research Foundation of South Africa (Grant Number: 123016).

## Conflict of interest

The authors declare that the research was conducted in the absence of any commercial or financial relationships that could be construed as a potential conflict of interest.

## Publisher’s note

All claims expressed in this article are solely those of the authors and do not necessarily represent those of their affiliated organizations, or those of the publisher, the editors and the reviewers. Any product that may be evaluated in this article, or claim that may be made by its manufacturer, is not guaranteed or endorsed by the publisher.
